# Prevalence of human respiratory syncytial virus infection in people with acute respiratory tract infections in Africa: A systematic review and meta‐analysis

**DOI:** 10.1111/irv.12584

**Published:** 2018-07-05

**Authors:** Sebastien Kenmoe, Jean Joel Bigna, Estelle Amandine Well, Fredy Brice N. Simo, Véronique B. Penlap, Astrid Vabret, Richard Njouom

**Affiliations:** ^1^ Department of Virology National Influenza Center Centre Pasteur of Cameroon Yaoundé Cameroon; ^2^ Department of Epidemiology and Public Health National Influenza Center Centre Pasteur of Cameroon Yaoundé Cameroon; ^3^ School of Public Health Faculty of Medicine University of Paris Sud Le Kremlin‐Bicêtre France; ^4^ Faculty of Medicine and Biomedical Sciences University of Yaoundé 1 Yaoundé Cameroon; ^5^ Department of Biochemistry Faculty of Sciences University of Yaoundé 1 Yaoundé Cameroon; ^6^ Normandie Université Caen France; ^7^ Groupe de Recherche sur l'Adaptation Microbienne (GRAM) Université de Caen Caen France; ^8^ Laboratoire de Virologie Centre Hospitalo‐Universitaire de Caen Caen France

**Keywords:** Africa, epidemiology, meta‐analysis, respiratory syncytial virus, respiratory tract infections

## Abstract

**Aim:**

The epidemiology of human respiratory syncytial virus (HRSV) infection has not yet been systematically investigated in Africa. This systematic review and meta‐analysis are to estimate the prevalence of HRSV infections in people with acute respiratory tract infections (ARTI) in Africa.

**Method:**

We searched PubMed, EMBASE, Africa Journal Online, and Global Index Medicus to identify observational studies published from January 1, 2000, to August 1, 2017. We used a random‐effects model to estimate the prevalence across studies. Heterogeneity (*I* ^2^) was assessed via the chi‐square test on Cochran's *Q* statistic. Review registration: PROSPERO CRD42017076352.

**Results:**

A total of 67 studies (154 000 participants) were included. Sixty (90%), seven (10%), and no studies had low, moderate, and high risk of bias, respectively. The prevalence of HRSV infection varied widely (range 0.4%‐60.4%). The pooled prevalence was 14.6% (95% CI 13.0‐16.4, *I* ^2^ = 98.8%). The prevalence was higher in children (18.5%; 95% CI 15.8‐21.5) compared to adults (4.0%; 95% CI 2.2‐6.1) and in people with severe respiratory tract infections (17.9%; 95% CI 15.8‐20.1) compared to those with benign forms (9.4%; 95% CI 7.4‐11.5); *P*‐values <0.0001. The HRSV prevalence was not associated with sex, subregion in Africa, setting, altitude, latitude, longitude, and seasonality.

**Conclusion:**

This study suggests a high prevalence of HRSV in people with ARTI in Africa, particularly among children and people with severe clinical form. All innovative strategies to curb the burden should first focus on children which present the highest HRSV‐related burden.

## 1 INTRODUCTION

Worldwide, acute respiratory tract infections (ARTI) are one of the leading causes of hospitalization, morbidity, and mortality among children under five.^1^ Globally, human respiratory syncytial virus (HRSV) is the most common pathogen identified in infants and young children with bronchiolitis and pneumonia. Based on published and unpublished studies, a global systematic and meta‐analysis by Shi and colleagues showed that an incidence of 33.1 (95% CI, 21.6‐50.3) million of cases of ARTI was associated with HRSV each year in children aged less than 5 years. A total of 3.2 (95% CI, 2.7‐3.8) million of these infections were severe cases requiring hospitalization. Approximately 40‐74.5 thousand deaths were estimated in children <5 with ARTI in this meta‐analysis.^2^ Another meta‐analysis had previously showed that the incidence of HRSV infection in developing countries was more than twice greater than that of developed countries and that 91% of hospitalizations and nearly all deaths (99%) were registered in developing countries where access to health care is limited.^3^ Preterm infants, those under 2 years, those over 65 years, and immunocompromised patients, are at higher risk of hospitalization for HRSV infections.^4^ Patients with a history of atopy, congenital heart disease, congestive heart failure, chronic obstructive pulmonary disease, neuronal and muscular disorders, and cancer are also at high risk for the development of severe HRSV infections.^5^ To date, there are more than 60 HRSV vaccine development programs at various stages. Some of them could be used in the next decade.

Africa, a continent in which most countries are developing with limited resources for health, the ecology in this continent can give it a particular epidemiology regarding HRSV infection. To the best of our knowledge, to date, no systematic review and meta‐analysis have been conducted in Africa on the epidemiology and drivers of HRSV infection. Therefore, we conducted a systematic review and meta‐analysis to estimate the prevalence and drivers of HRSV infection among people with ARTI in Africa. We have done this work to provide accurate data to guide health policymakers and to identify information gaps to guide future research.

## 2 MATERIAL AND METHODS

### 2.1 Search strategy and selection criteria

We performed a comprehensive and exhaustive search of MEDLINE through PubMed, Excerpta Medica Database (EMBASE), Africa Journals Online, and Global Index Medicus to identify all relevant articles published on HRSV in Africa from January 1, 2000, until August 31, 2017, regardless of language of publication. Both text words and medical subject heading terms were used. The following terms and their variants were used for HRSV: “HRSV,” “human respiratory syncytial virus,” and “respiratory syncytial virus.” Individual country names for the 54 African countries and African subregion names such as “Northern Africa” or “Southern Africa” were also used as additional key search terms for more abstracts on the subject. African country names were introduced both in English and in languages relevant to each country, for example, “Ivory Coast” and “Côte d'Ivoire”. Where country names have changed over time, old and new names were included, such as “Zaire” and “Democratic Republic of Congo”. Titles and abstracts of all eligible papers were reviewed, and full texts of articles were accessed. Search strategy conducted in PubMed is shown in Table S1. This search strategy was adapted to fit with other databases. A manual search which consists in scanning the reference lists of eligible papers and other relevant review articles was conducted. The search in electronic databases was conducted on September 6, 2017.

We considered observational studies (cross‐sectional, case‐control, and cohort). In the case of duplicate reports, the most comprehensive/complete and up‐to‐date version was considered. We considered studies including patients with clinical diagnosis of acute respiratory tract infection as defined in each study. Studies among populations with underlying medical conditions, studies conducted during an outbreak period, case series or studies in which HRSV was imported cases were excluded. The search for HRSV had to be conducted systematically or by sampling of the population in the presence of defined inclusion criteria (respiratory signs) and HRSV detection by polymerase chain reaction (PCR) technique on respiratory samples. Studies lacking or with not extractable primary data and/or explicit method description were excluded. In the case of missing data, we contacted authors of the paper. We planned to use Google Translate in the case of other languages than French, English, or Spanish.

Two investigators independently screened records based on titles and abstracts for eligibility. Full texts of articles deemed potentially eligible were retrieved. Further, these investigators independently assessed the full text of each study for eligibility and consensually retained studies to be included. Disagreements when existing were solved through a discussion.

### 2.2 Data extraction and management

Data were extracted using a preconceived and tested data abstraction form. In the cases of missing data, authors were directly contacted to provide missing information. Two investigators independently extracted data including name of the first author, publication year, study design, setting, sampling method, respiratory samples collection period, timing of data analysis, number of viruses screened, site of recruitment location (country, city, latitude, longitude, and altitude), clinical presentation, number of patients screened, number of patients infected with HRSV, diagnostic techniques used, and proportion of male participants. We assigned a United Nations Statistics Division (UNSD) African region (central, eastern, northern, southern, and western) to each study regarding the country of recruitment.^6^ We considered two groups of clinical presentation; severe respiratory tract infection (SRTI) including severe acute respiratory infection, acute lower respiratory infections, bronchitis, bronchiolitis, pneumonia, severe or very severe pneumonia, and benign respiratory tract infection (BRTI) including upper respiratory tract infection, and influenza‐like illness. Using Google Global Positioning System, we assigned altitude, latitude, and longitude according to the cities and country of recruitment.^7^ In the case of multicities, we considered the median. Disagreements between investigators were reconciled through discussion and consensus or an arbitration of a third investigator.

Two investigators evaluated risk of bias in included studies using an eight‐item rating scale.^8^ These items included (a) participation response rate more than 75% agree to participate or analysis to show whether respondents and nonrespondents were similar for the sociodemographic characteristics; (b) ARTI clearly defined; (c) method of inclusion identical for all subjects; (d) description of diagnostic technique; (e) same type of sample collected for all patients (nasopharyngeal aspirate, nasal, or throat swab); (f) standardized method for sample collection (quantity of aspirate or of liquid used for the nasal wash with any virological medium transport for swabs); (g) analysis performed according to relevant subgroups (by age classes, by center, or by symptomatology, for example); and (h) and presentation of data sources (counts are presented, not only percentages). Each item was assigned a score of 1 (Yes) or 0 (No), and each score was summed across items to generate an overall study quality score. The total score was ranged from 0 to 8 with the overall score categorized as follows: 6‐8: “low risk of bias,” 3‐5: “moderate risk,” and 0‐2: “high risk.” Disagreements were solved through discussion and consensus.

### 2.3 Data synthesis and analysis

Data analyses used the “meta” and “metafor” packages of the statistical software r (version 3.3.3, The R Foundation for statistical computing, Vienna, Austria). Unadjusted prevalence and standard errors of HRSV infection were recalculated based on the information of crude numerators and denominators provided by individual studies. To keep the effect of studies with extremely small or extremely large prevalence estimates on the overall estimate to a minimum, the variance of the study‐specific prevalence was stabilized with the Freeman‐Tukey double‐arcsine transformation before pooling the data within a random‐effects meta‐analysis model.^9^ Symmetry of funnel plot and Egger's test served to assess the presence of publication bias.^10^ A *P*‐value <0.10 on Egger test was considered indicative of statistically significant publication bias.

Heterogeneity was evaluated by the chi‐square test on Cochrane's *Q* statistic,^11^ which was quantified by *H* and *I* ^2^ values. The *I* ^2^ statistic estimates the percentage of total variation across studies due to true between‐study differences rather than chance. In general, *I* ^2^ values greater than 60%‐70% indicate the presence of substantial heterogeneity. The value of *H* close to 1 is indicative of some homogeneity between studies.^12^ Subgroup analyses were performed for the following subgroup: age groups (0‐5/>5 years, children/adults), sex, clinical presentation, setting, hemisphere (north/south), location according to Greenwich meridian (East/West), by median altitude, and UNSD African Regions. Univariable and multivariable meta‐regressions were used to test for an effect of study and participants’ characteristics. To be included in multivariable meta‐regression analysis, a *P* value <0.25 in univariable analysis was considered. For categorical variables, the global *P* value was considered for the inclusion in multivariable models. A *P* value <0.05 was considered statistically significant. We reported the explained heterogeneity (*R*²) for the residual heterogeneity of HRSV prevalence. Following crude overall prevalence, two sensitivity analyses were conducted: one considering only studies with low risk of bias and another one considering only studies conducted in complete season(s).

The Centre for Reviews and Dissemination guidelines was used for the methodology of this review.^13^ The Preferred Reporting Items for Systematic Reviews and Meta‐Analyses guidelines served as the template for reporting the present review (Table S2).^14^ This review is registered in the PROSPERO International Prospective Register of Systematic Reviews, registration number CRD42017076352.

## 3 RESULTS

### 3.1 Review process

**Figure 1:**
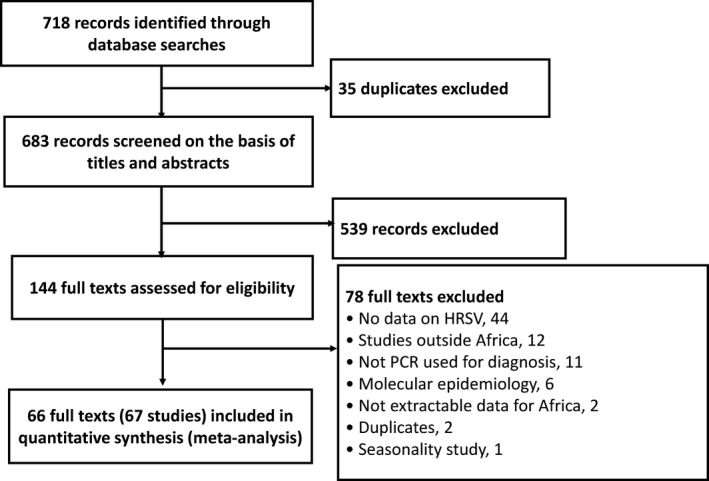
Review process

We identified 718 records; after elimination of duplicates, 683 records remained. After screening of titles and abstracts, we found 539 records to be irrelevant and excluded them. We assessed full texts of the remaining 144 papers for eligibility, of which 78 were excluded with reasons (Figure 1). The inter‐rater agreement for study selection was high (*κ* = 0.81). Finally, 66 full texts including 67 studies were included (one paper included two studies).^15^, ^16^, ^17^, ^18^, ^19^, ^20^, ^21^, ^22^, ^23^, ^24^, ^25^, ^26^, ^27^, ^28^, ^29^, ^30^, ^31^, ^32^, ^33^, ^34^, ^35^, ^36^, ^37^, ^38^, ^39^, ^40^, ^41^, ^42^, ^43^, ^44^, ^45^, ^46^, ^47^, ^48^, ^49^, ^50^, ^51^, ^52^, ^53^, ^54^, ^55^, ^56^, ^57^, ^58^, ^59^, ^60^, ^61^, ^62^, ^63^, ^64^, ^65^, ^66^, ^67^, ^68^, ^69^, ^70^, ^71^, ^72^, ^73^, ^74^, ^75^, ^76^, ^77^, ^78^, ^79^, ^80^

### 3.2 Characteristics of included studies

Sixty (90%), seven (10%), and no studies had low, moderate, and high risk of bias, respectively. Studies were published between 2010 and 2017. Data were from 20 countries. Respiratory samples were collected between January 2006 and April 2016. Most of data came from cross‐sectional studies, used consecutive sampling method, and prospective design. Most of studies included only children with proportion of males varying between 44.1% and 69.8%. Real‐time reverse transcription‐polymerase chain reaction was the most used diagnosis method. Most of studies was conducted in both urban and rural settings and in Eastern Africa region (Table 1). Individual characteristics of included studies are in the Table S3.

**Table 1:** Characteristics of included studies

Characteristics	Data
Design, n (%)	
Cross‐sectional	63 (94)
Case‐control	4 (6)
Setting, n (%)	
Rural	12 (18)
Urban	10 (15)
Urban and rural	29 (43)
Not described	16 (24)
Sampling method, n (%)	
Consecutive	57 (85)
Systematic	3 (4)
Random	6 (9)
Timing, n (%)	
Prospective	64 (96)
Retrospective	3 (4)
Clinical presentation	
BRTI	9 (13)
SRTI	38 (57)
BRTI and SRTI	20 (30)
Population, n (%)	
Children	39 (58)
Adults	2 (3)
Children and adults	26 (39)
Region, n (%)	
Eastern (Kenya = 17; Madagascar = 2; Malawi = 1; Mozambique = 2; Reunion = 1)	22 (33)
Southern (Botswana = 1; Malawi = 1; Mozambique = 1; South Africa = 12; Zambia = 1)	15 (22)
Northern (Egypt = 10; Morocco = 3; Sudan = 1)	14 (21)
Western (Burkina Faso = 2; Côte d'Ivoire = 1; Ghana = 2; Niger = 1; Nigeria = 1; Senegal = 5)	12 (18)
Central (Cameroon = 2; Gabon = 1; Central Africa Republic = 1)	4 (6)
Diagnostic technique, n (%)	
Real‐time RT‐PCR	64 (96)
Conventional RT‐PCR	3 (4)
Latitude, median (1st; 3rd quartiles), in decimal degrees	3.5 (−9.4; 15.0)
Longitude, median (1st; 3rd quartiles), in decimal degrees	30.5 (11.8‐34.4)
Altitude, median (1st; 3rd quartiles), in meters	252 (28.5‐1012.5)

### 3.3 Prevalence of HRSV infection among ARI in Africa

**Figure 2:**
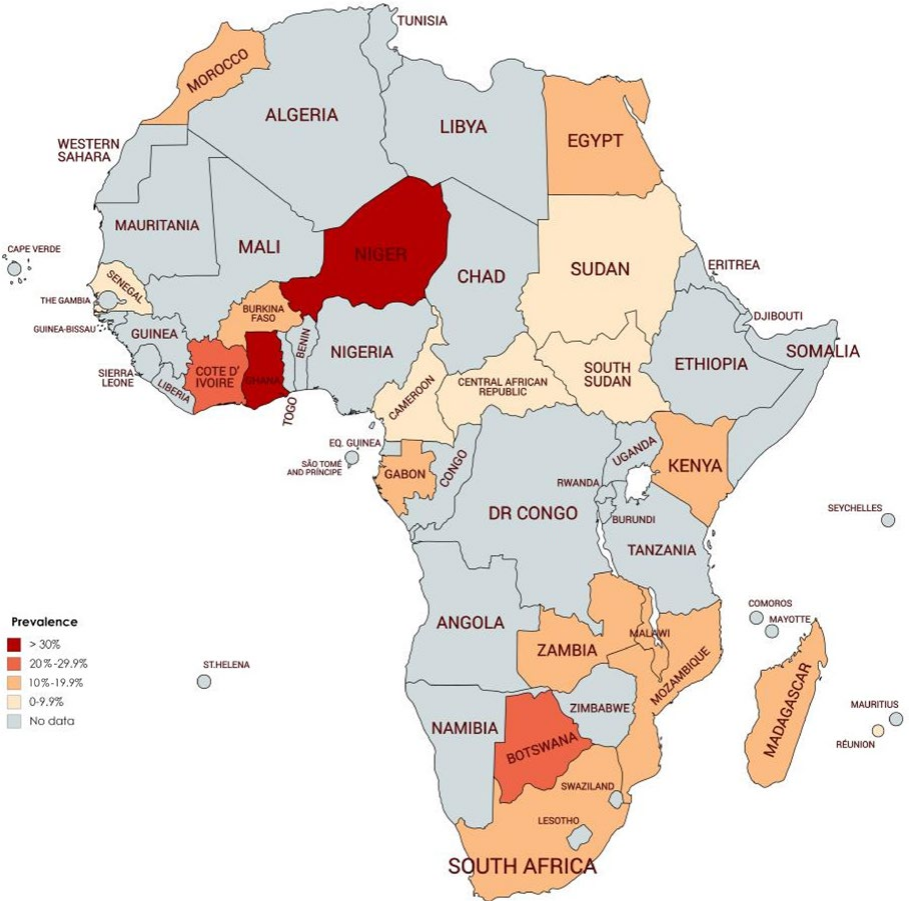
Distribution of prevalence of Human Respiratory Syncytial virus infection among patients with ARI in Africa continent

**Figure 3:**
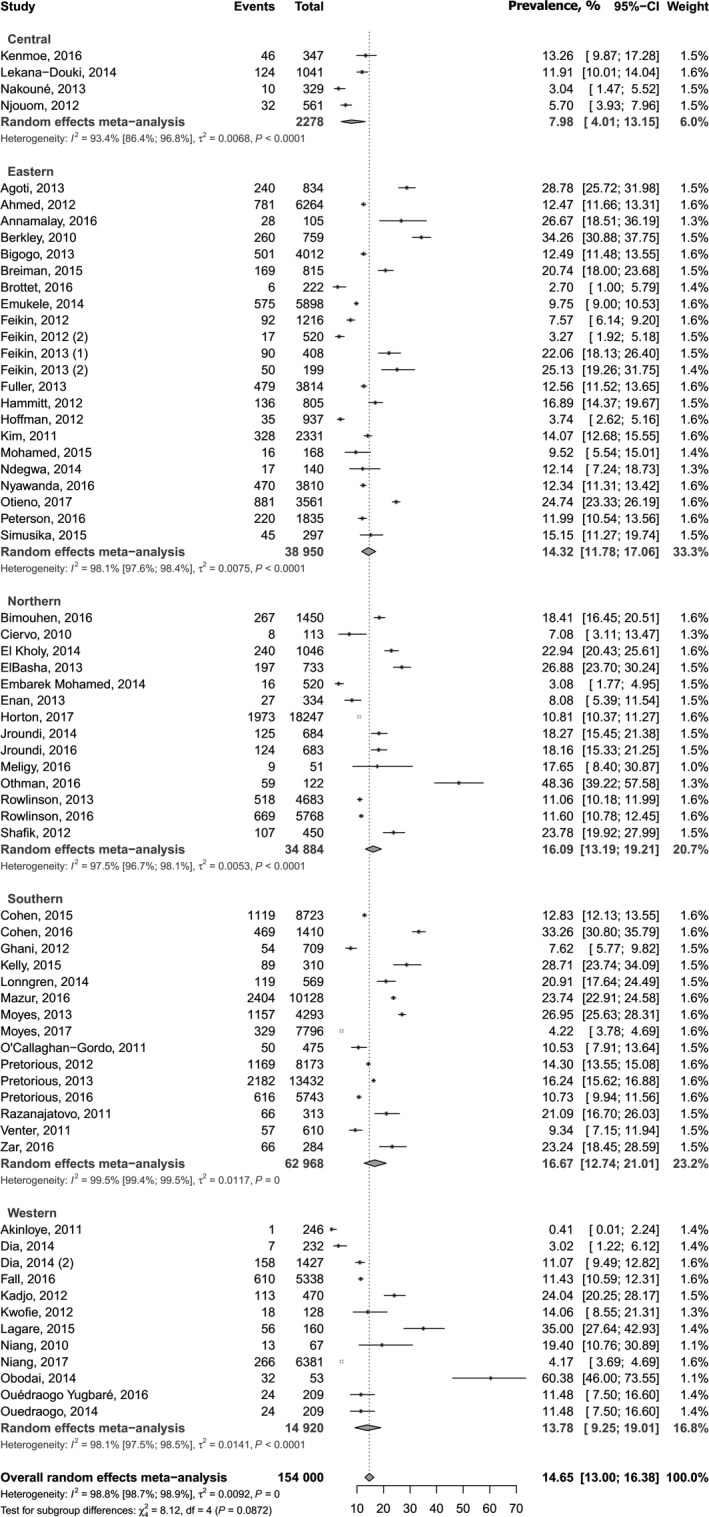
Meta‐analysis results for the prevalence of human respiratory syncytial virus prevalence in Africa

Table 2 summarizes results from meta‐analyses. The prevalence varied widely from 0.4% to 60.4% across countries. The distribution of the prevalence was not uniform in the continent (Figure 2). The overall prevalence was 14.6% (95% CI 13.0‐16.4) in a pooled sample of 154 000 participants with ARTI (Figure 3). The funnel plot did not suggest any publication bias (Figure S1), and this result is confirmed by the Egger test (Table 2). The prevalence in studies with low risk of bias was not different to the overall prevalence (Table 2). Substantial heterogeneity was present for overall and within all subgroups (Table 2). Publication bias was found for the following subgroup analyses: SRTI, North Hemisphere, and Northern Region of Africa (Table 2).

**Table 2:** Summary and comparison statistics of human respiratory syncytial virus prevalence in Africa

Groups	N studies	N participants	Prevalence, % (95% confidence interval)	*I* ^2^ (95% confidence interval	*H* (95% confidence interval)	*P* heterogeneity	*P* Egger test	*P* difference
Overall	67	154 000	14.6 (13.0‐16.4)	98.8 (98.7‐98.9)	9.1 (8.7‐9.5)	<0.0001	0.298	‐
Complete season(s) studies	40	82 048	13.6 (11.2‐16.2)	99.0 (98.9‐99.1)	10.0 (9.4‐10.6)	<0.0001	0.148	‐
Low risk of bias studies	60	151 017	14.3 (12.6‐16.1)	98.9 (98.7‐99.0)	9.3 (8.9‐9.8)	<0.0001	0.404	‐
Subgroup analyses								
Age group								
Children (0‐15 y)	34	39 984	18.5 (15.8‐21.5)	98.0 (97.6‐98.3)	7.0 (6.5‐7.6)	<0.0001	0.512	<0.0001
Adults	5	11 976	4.0 (2.2‐6.1)	94.6 (90.2‐97.0)	4.3 (3.2‐5.8)	<0.0001	0.904	
Age group								
0‐5 y	19	29 843	22.6 (18.8‐26.6)	98.2 (97.8‐98.6)	7.5 (6.8‐8.3)	<0.0001	0.747	<0.0001
>5 y	8	15 171	6.0 (3.8‐8.6)	96.0 (94.0‐97.4)	5.0 (4.1‐6.2)	<0.0001	0.134	
Clinical presentation								
SRTI	45	118 083	17.9 (15.8‐20.1)	98.8 (98.6‐98.9)	9.1 (8.6‐9.6)	<0.0001	0.033	<0.0001
BRTI	20	30 472	9.4 (7.4‐11.5)	97.0 (96.2‐97.6)	5.7 (5.1‐6.5)	<0.0001	0.367	
Settings								
Rural	10	16 973	14.1 (10.4‐18.2)	97.8 (96.9‐98.3)	6.7 (5.7‐7.8)	<0.0001	0.308	0.402
Urban	10	7150	16.6 (12.5‐21.1)	95.6 (93.6‐97.0)	4.8 (3.0‐5.8)	<0.0001	0.462	
Sex								
Male	2	755	31.1 (9.8‐57.8)	94.2 (81.8‐98.2)	4.2	<0.0001	NA	0.948
Female	2	817	32.6 (5.3‐68.8)	96.8 (91.6‐98.8)	5.6	<0.0001	NA	
Hemisphere								
North	38	75 069	14.6 (12.5‐16.9)	98.5 (98.2‐98.7)	8.1 (7.5‐8.6)	<0.0001	0.078	0.960
South	29	78 931	14.7 (12.2‐17.4)	99.0 (98.9‐99.1)	10.0 (9.4‐10.7)	<0.0001	0.991	
Meridian								
East	56	137 481	14.6 (12.9‐16.5)	98.8 (98.7‐98.9)	9.1 (8.6‐9.5)	<0.0001	0.474	0.886
West	11	16 519	14.9 (10.3‐20.3)	98.4 (97.9‐98.8)	7.9 (7.0‐9.1)	<0.0001	0.096	
Regions								
Central	4	2278	8.0 (4.0‐13.1)	93.4 (86.4‐96.8)	3.9 (2.7‐5.6)	<0.0001	0.488	0.087
Eastern	22	38 950	14.3 (11.8‐17.1)	98.1 (97.6‐98.4)	7.2 (6.5‐7.9)	<0.0001	0.545	
Northern	14	34 884	16.1 (13.2‐19.2)	97.5 (96.7‐98.1)	6.3 (5.5‐7.2)	<0.0001	0.053	
Southern	15	62 968	16.7 (12.7‐21.0)	99.5 (99.4‐99.5)	13.5 (12.5‐14.6)	<0.0001	0.693	
Western	12	14 920	13.8 (9.2‐19.0)	98.1 (97.5‐98.5)	7.3 (6.4‐8.3)	<0.0001	0.162	

BRTI, benign respiratory tract infection; NA, not applicable; RT‐PCR, reverse transcription‐polymerase chain reaction; SRTI, severe respiratory tract infection.

### 3.4 Subgroup analyses

The prevalence was higher among children (18.5%, 95% CI 15.8‐21.5) compared to adults (4.0%, 95% CI 2.2‐6.1; *P* < 0.0001), among people aged <5 years (22.6%, 95% CI 18.8‐26.6) compared to those aged >5 years (6.0%, 95% CI 3.8‐8.6; *P* < 0.0001), and among people with SRTI (17.9%, 95% CI 15.8‐20.1) compared to those with BRTI (9.4%, 95% CI 7.4‐11.5; *P* < 0.0001) (Table 2). There was no difference for UNSD subregions of Africa, sex, setting, hemisphere, and distribution by Greenwich meridian (Table 2).

### 3.5 Factors associated with prevalence of HRSV infections

In univariable meta‐regression analysis, the prevalence was associated with clinical presentation (*R* ^2^ = 8.99%) and age groups (*R* ^2^ = 38.84%). In multivariable meta‐regression analysis, the prevalence was higher in children compared to adults (adjusted odds ratio: 1.28, 95% CI: 1.09‐1.52, *P* = 0.0041). Variables included in the multivariable model (clinical presentation, age groups, diagnostic method) explained 42.8% of the 97.6% residual heterogeneity of HRSV prevalence (Table S4).

## 4 DISCUSSION

This systematic review and meta‐analysis of data from 67 studies involving 154 000 individuals found a pooled prevalence of HRSV infection of 14.6% in people with ARTI in Africa, with substantial heterogeneity. Sensitivity analysis including only studies with low risk of bias and another one including only studies conducted in complete seasons gave similar results. The drivers of high prevalence of HRSV infection included being children and having SRTI.

The prevalence of HRSV infection in people with ARTI from Africa found in this review is close to that found in other systematic reviews from another place. A systematic review with meta‐analysis in China found a prevalence of 18.7% (95% CI 17.1‐20.5, 135 studies from 2010 to 2015) attributable to HRSV.^81^ Another systematic review with meta‐analysis of 21 studies (1996‐2013) in Iran yielded to a prevalence of 18.7%.^82^ Another meta‐analysis including participants with lower ARTI from Latin America found a prevalence varying from 40.9% among children aged less than 1 year to 12.6% adults aged more than 65 years.^83^ One should note that these three systematic reviews included studies using less sensitive and less specific immunofluorescence and immune‐chromatographic techniques. Our review included studies using only reverse transcriptase PCR. The interpretation of the prevalence found in our study should consider the involvement of other infectious agents in the occurrence of ARTI. Indeed, the presence of coinfection with other viruses should be considered. These coinfections may have a synergistic effect in the occurrence of ARTI. Similarly, bacterial superinfection that may favor clinical presentation in the form of SRTI should also be considered. Thus, this prevalence of 14.6% in our study could not represent the proportion of ARTI cases attributable to HRSV alone, but its implication on the occurrence of ARTI in people from Africa. However, this hypothesis, involvement of bacterial superinfection or viral coinfection on the occurrence of STRI in the presence of HRSV infection, needs to be investigated in futures studies.

We found in this review that the prevalence of HRSV infection was higher in children compared to adults. This is consistent with other reviews.^4^, ^5^, ^81^, ^82^, ^83^ Similar data were found in developed countries.^84^ This means that the etiologies of ARTI including viruses and other agents such as bacteria are not the same between adults and children. Indeed, in the published literature, HRSV infections appear as one of the major cause of mortality and morbidity including causes of hospitalizations in children and not among adults.^85^, ^86^, ^87^ Children may have a naturally weak immune system compared to adults when facing HRSV infection.^88^ Innovative strategies to curb the burden should first focus on children which present the highest HRSV‐related burden.^2^ Effective and efficient maternal HRSV vaccine and monoclonal antibody use may help to curb the disease burden in this vulnerable population. In fact, an effective maternal immunization or if newborn antibody immunization is successful,^89^, ^90^ it is possible to decrease by 80% the involvement of HRSV in the occurrence of ARTI in children.^2^, ^91^

We found a higher prevalence of HRSV infection in SRTI compared to BRTI patients. A review in China reported a higher prevalence in inpatients compared outpatients.^81^ SRTI clinical presentation may mainly due to the coinfection with bacteria; therefore, this clinical presentation could reflect the consequence of a superinfection of HRSV infection. We did not find any association between prevalence and altitude, latitude, and longitude suggesting that these parameters do not influence the distribution of HRSV throughout the Africa continent. In addition, there was no difference in distribution across Africa subregions. One would have expected that the increasing distance from the equator or in case of the increasing altitude, one can have an increase of the prevalence.

Results from this study should be interpreted with caution in the context of its limitations. First, we found substantial heterogeneity in estimation of the prevalence of HRSV infection in people with ARI across studies. Although we identified some sources of heterogeneity, there may still be other sources of heterogeneity not investigated. These other sources of heterogeneity could include environmental temperature, humidity, pluviometry, exposure to smoking, and air pollution. However, we were unable to assess these factors as source of heterogeneity because they were not reported in primary included studies. Second, countries and UNSD African regions were not uniformly represented, partly owing to difficult retrieval of African medical literature, especially for older articles and those published in local journals. This can limit the generalizability of findings to the entire African continent. Thirdly, even though we aimed at including all age groups, most of studies were conducted in pediatric populations. Our findings therefore mainly reflect the prevalence of HRSV infection in children with ARI.

To the best of our knowledge, this article is among the first systematic reviews that use meta‐analysis to summarize data on prevalence of HRSV infection in people with ARI in Africa. Strengths of this systematic review and meta‐analysis include the use of predefined protocol, a comprehensive search strategy, and involvement of two independent investigators in all stages of the review process. We included only studies that identified HRSV using a reference standard method for diagnosis, the reverse transcriptase PCR. Although we found publication bias in some subgroup analyses, no publication bias was found in the main analyses suggesting that we were unlikely to have missed studies that could alter the findings. Nine‐tenths of the studies were assessed as having low risk of bias in their methodological quality, suggesting that we can be confident to the quality of the findings in this review. In addition, the sensitivity analyses including only studies with low risk and studies with complete season(s) yielded to very close prevalence to that in the crude analysis. A multivariable meta‐regression analysis was conducted helping to control potential confounders for source of variation in HRSV prevalence.

This study suggests a high prevalence of HRSV in people with ARTI in Africa, particularly among children and people with severe clinical form presentation. As such, HRSV infections in people with ARTI in Africa should deserve more attention from healthcare providers, researchers, policymakers, and stakeholders from the health sector for improved detection, overall proper management, and efficient control. Actual influenza surveillance systems implemented in Africa could add routine screening of HRSV in collected samples. Efforts to address this burden could mainly focus on primary prevention including development and implementation of vaccines against HRSV. Strategies and funding to expand the use of vaccines and other primary prevention methods in this resource‐constrained continent are needed. All innovative strategies to curb the burden should first focus on children which present the highest HRSV‐related burden.

## CONFLICT OF INTERESTS

The authors declare that they have no competing interests.

## DATA SHARING AND DATA ACCESSIBILITY

All data generated or analyzed during this study are included in this published article and its supplementary information files.

## AUTHOR'S CONTRIBUTIONS

SK, JJB, and RN involved in conception and design. SK and JJB involved in literature search.SK and JJB involved in study selection. SK, JJB, FBNS, and EAW extracted the data. JJB and SK involved in data synthesis and analysis.SK and JJB made the first draft. SK, JJB, EAW, FBNS, VBP, AV, and RN involved in critical revision of successive drafts of the paper. RN is guarantor of the review. All authors approved the final version.
