# Supplementary material for: Prevalence of human respiratory syncytial virus infection in people with acute respiratory tract infections in Africa: A systematic review and meta‐analysis
Source: Influenza Other Respir Viruses. 2018 Jul 5;12(6):793–803. doi: 10.1111/irv.12584 (PMC6185896; doi:10.1111/irv.12584)
Supplement: Supplementary file 2 [file IRV-12-793-s002.docx]

**Table S1. Search strategy and results on MEDLINE through PubMed**

| **Search** | **Search terms** |
| --- | --- |
| #1 | “HRSV” OR “RSV” OR “human respiratory syncytial virus” OR “respiratory syncytial virus” |
| #2 | “respiratory tract infections” OR “respiratory tract infection” OR “respiratory infection” OR “respiratory infections” OR “lower respiratory tract infections” OR “LRTI” OR “acute lower respiratory infections” OR “ALRI” OR “pneumonia” OR “community acquired pneumonia” OR “bronchiolitis” OR “severe acute respiratory infections” OR “severe acute respiratory illness” OR “experimental lung inflammation” OR “pneumonitis” OR “pulmonary inflammation” OR “bronchopneumonia” OR “pleuropneumonia” |
| #3 | Africa* OR Algeria OR Angola OR Benin OR Botswana OR "Burkina Faso" OR Burundi OR Cameroon OR "Canary Islands" OR "Cape Verde" OR "Central African Republic" OR Chad OR Comoros OR Congo OR "Democratic Republic of Congo" OR Djibouti OR Egypt OR "Equatorial Guinea" OR Eritrea OR Ethiopia OR Gabon OR Gambia OR Ghana OR Guinea OR "Guinea Bissau" OR "Ivory Coast" OR "Cote d'Ivoire" OR Jamahiriya OR Kenya OR Lesotho OR Liberia OR Libya OR Madagascar OR Malawi OR Mali OR Mauritania OR Mauritius OR Mayotte OR Morocco OR Mozambique OR Namibia OR Niger OR Nigeria OR Principe OR Reunion OR Rwanda OR "Sao Tome" OR Senegal OR Seychelles OR "Sierra Leone" OR Somalia OR "South Africa" OR “South Sudan” OR "St Helena" OR Sudan OR Swaziland OR Tanzania OR Togo OR Tunisia OR Uganda OR "Western Sahara" OR Zaire OR Zambia OR Zimbabwe OR "Central Africa" OR "Central African" OR "West Africa" OR "West African" OR "Western Africa" OR "Western African" OR "East Africa" OR "East African" OR "Eastern Africa" OR "Eastern African" OR "North Africa" OR "North African" OR "Northern Africa" OR "Northern African" OR "South African" OR "Southern Africa" OR "Southern African" OR "sub Saharan Africa" OR "sub Saharan African" OR "sub Saharan Africa" OR "sub Saharan African” |
| #4 | #1 AND #2 AND #3 |
| #5 | Limits 2000/01/01-2017/08/31 |
