# Supplementary material for: Prevalence of human respiratory syncytial virus infection in people with acute respiratory tract infections in Africa: A systematic review and meta‐analysis
Source: Influenza Other Respir Viruses. 2018 Jul 5;12(6):793–803. doi: 10.1111/irv.12584 (PMC6185896; doi:10.1111/irv.12584)
Supplement: Supplementary file 5 [file IRV-12-793-s005.docx]

**Table S4. Univariable and multivariable meta-regressions of the prevalence Human Respiratory Syncytial Virus infections in people with acute respiratory infections in Africa**

| **Variables (reference)** | **Univariable analysis** | | | **Multivariable analysis^¥^** | |
| --- | --- | --- | --- | --- | --- |
|  | **Coefficient (95% confidence interval)** | **P value** | **R²** | **Coefficient (95% confidence interval)** | **P value** |
| **Clinical presentation (BRTI)** |  | 0.0003 | 8.99 |  |  |
| SRTI | 0.1360 (0.0439 ; 0.2281) |  |  | -0.0319 (-0.1244 ; 0.0605) | 0.2594 |
| SRTI and BRTI | 0.0064 (-0.0934 ; 0.1062) |  |  | 0.0543 (-0.0411 ; 0.1498) | 0.4922 |
| **Year of publication** | 0.0094 (-0.0083 ; 0.0271) | 0.2916 | 0.00 |  |  |
| **Seasonality (complete season(s))** |  | 0.2693 | 1.42 |  |  |
| Not complete season(s) | 0.0390 (-0.0309 ; 0.1088) |  |  |  |  |
| **Age group (Adults)** |  | < 0.0001 | 38.84 |  |  |
| Children | 0.2627 (0.0953 ; 0.4302) |  |  | 0.2497 (0.0821 ; 0.4173) | 0.0041 |
| Children and adults | 0.1231 (-0.0460 ; 0.2923) |  |  | 0.1558 (-0.0120 ; 0.3237) | 0.0683 |
| **Setting (rural)** |  | 0.5340 | 0.00 |  |  |
| Urban | 0.0542 (-0.0534 ; 0.1619) |  |  |  |  |
| Urban and rural | 0.0072 (-0.0789 ; 0.0933) |  |  |  |  |
| **Sampling method (consecutive)** |  | 0.5164 | 0.00 |  |  |
| Random | 0.1015 (-0.0743 ; 0.2774) |  |  |  |  |
| Systematic | 0.0006 (-0.1103 ; 0.1115) |  |  |  |  |
| **Sample size** | -0.0000 (-0.0000 ; 0.0000) | 0.3581 | 0.00 |  |  |
| **Score on risk of bias** | 0.0015 (-0.0243 ; 0.0274) | 0.9072 | 0.51 |  |  |
| **Diagnostic method (conventional RT-PCR)** |  |  |  |  |  |
| Real time RT-PCR | -0.1415 (-0.3204 ; 0.0373) | 0.1188 | 0.00 | -0.0720 (-0.2271 ; 0.0831) | 0.3568 |
| **Timing of data collection (prospective)** |  |  | 0.00 |  |  |
| Retrospective | -0.0757 (-0.2422 ; 0.0908) | 0.3672 |  |  |  |
| **Latitude** | -0.0002 (-0.0019 ; 0.0015) | 0.8264 | 0.00 |  |  |
| **Absolute latitude** | 0.0013 (-0.0014 ; 0.0040) | 0.3330 | 0.00 |  |  |
| **Longitude** | 0.0002 (-0.0018 ; 0.0022) | 0.8540 | 2.34 |  |  |
| **Altitude** | -0.0000 (-0.0001 ; 0.0000) | 0.3417 | 0.00 |  |  |
| **Regions (Central)** |  | 0.5080 | 0.00 |  |  |
| Eastern | 0.1014 (-0.0508 ; 0.2537) |  |  |  |  |
| Northern | 0.1280 (-0.0312 ; 0.2872) |  |  |  |  |
| Southern | 0.1330 (-0.0242 ; 0.2902) |  |  |  |  |
| Western | 0.0914 (-0.0718 ; 0.2546) |  |  |  |  |

RT-PCR: reverse transcriptase polymerase chain reaction, SRTI: severe respiratory tract infection; BRTI: benign respiratory tract infection

**¥:** Residual heterogeneity I² = 97.6%; Explained heterogeneity by variables included in the model R² = 42.8%
